# 18-Fluorodeoxyglucose Positron Emission Tomography-Computed Tomography to Guide Aortic Arch Replacement in Relapsing Polychondritis

**DOI:** 10.1055/s-0039-1687903

**Published:** 2019-07-22

**Authors:** Raymond Pfister, Maria Messe, Lars Niclauss, Matthias Kirsch, Claude Haller, Dominique Delay

**Affiliations:** 1 Department of Cardiovascular Surgery, University Hospital of Lausanne (CHUV), Lausanne, Switzerland; 2 Department of Cardiac Surgery, Valais Hospital, Sion, Switzerland; 3 Department of Neurology, Valais Hospital, Sion, Switzerland; 4 Department of Vascular Surgery, Valais Hospital, Sion, Switzerland

**Keywords:** relapsing polychondritis, vasculitis, PET–CT, aorta, aortic arch replacement

## Abstract

Relapsing polychondritis (RP) is a rare progressive autoimmune disease. The cardiovascular system is rarely involved. The authors report the case of a young woman with RP aortic arch aneurysm and symptomatic cerebral vessels stenosis. A positron emission tomography-computed tomography (PET–CT) indicated areas with activity and guided the surgery. Aortic arch with proximal vessels was successfully replaced. The PET–CT may be useful to assess the risks and determine healthy zones for potential anastomotic sites.

## Introduction

Relapsing polychondritis (RP) is, with an incidence of 3.5 per million/year, a rare, severe, episodic, and progressive inflammatory condition involving predominantly cartilaginous structures, mainly the ears, nose, and the laryngotracheobronchial tree. The etiology of RP is still unknown, but an autoimmune Th1 cell-mediated systemic inflammatory response may be one main mechanism. The clinical presentation may appear similar to other rheumatic and autoimmune diseases. 1 2 Generally observed in the fourth and fifth decades of life, RP incidences are equal for both sexes. The cardiovascular system, particularly the aortic and mitral valves, may be involved in up to 2 to 6% of patients. 1

## Case Presentation

A 29-year-old woman was admitted with fluctuating paresthesia of the right arm, hyposensitivity of the right cheek during 1 week, and an episode of blurred vision of the left eye.

An RP was diagnosed 5 years earlier, mainly based on combined scleritis, arthritis, and chondritis of both ears and her nose. An asymptomatic aortic arch dilatation was found incidentally 9 months before on a computed tomography (CT) scan, adjacent to a thickening of the innominate artery (IA) and an occlusion of the left common carotid artery (LCCA). Current medical treatment consisted of Actemra (anti-interleukine-6R) 162 mg per week, Simponi (anti-tumor necrosis factor α) 50 mg monthly, aspirin 100 mg per day, and methotrexate 10 mg per week.

The patient had no prior neurological complaints.

On admission, blood pressure was 96/77 mm Hg, heart rate was regular at 77 beats per minute, blood oxygen saturation at 98%, and body temperature was 36.6°C. Neurological examination was, apart from a transient hyposensitivity of the right upper limb, normal. Neuroimaging by magnetic resonance imaging did not indicate any signs of ischemic cerebral infarction or intracranial bleeding.

Ultrasound examination of the major supra-aortic vessels revealed an aneurysmal dilatation of the IA, a subtotal occlusion of the right common carotid artery (RCCA), the right subclavian artery (RSA), and the left subclavian artery (LSA) as well as the already known occlusion of the LCCA. Blood flow to the left internal carotid artery was conserved via retrograde perfusion from the left external carotid via intraophthalmic collaterals. The circle of Willis was complete.

**Fig. 1:**
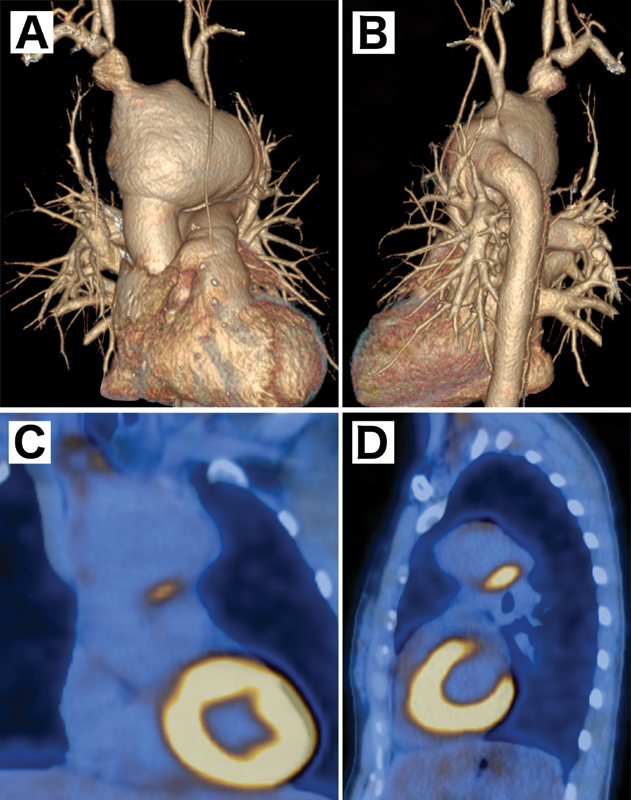
( **A** , **B** ) Computed tomography scan reconstruction showing aortic arch aneurysm (65 mm), innominate artery aneurysm, severe stenosis of the right subclavian artery, right common carotid artery, left subclavian artery, and occlusion of the left common carotid artery. ( **C** , **D** ) Fludeoxyglucose uptake in the aortic arch and innominate artery.

A new angio CT scan showed an ongoing dilatation of the aortic arch from 45 to 65 mm and confirmed the aforementioned supra-aortic vessel lesions ( Fig. 1 ).

To evaluate the activity of the vasculitis, we performed a positron emission tomography (PET)-CT. Increased contrast uptake was solely localized at the aneurysmal portion of the aortic arch and the IA ( Fig. 1 ), indicating an active inflammatory process responsible for the acute vessel dilatation.

Open surgical arch and proximal supra-aortic vessel replacement was considered as the treatment of choice. The reasons for surgery were, first, because the patient had symptoms from her stenotic lesions and, second, because of the risk of dissection in the ascending aorta and the arch. Another point is that even under maximal treatment concerning her RP, the disease was still progressing.

**Fig. 2:**
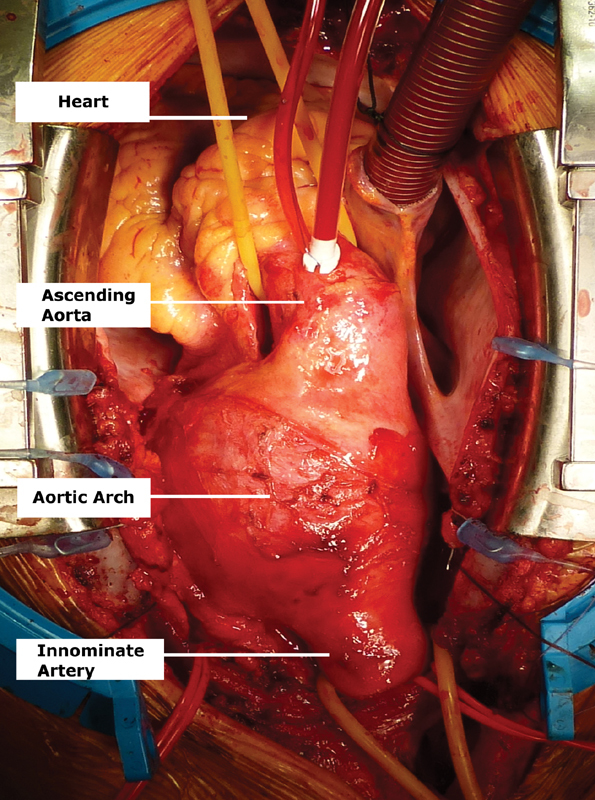
Intraoperative view of the aortic arch and innominate aneurysms.

**Fig. 3:**
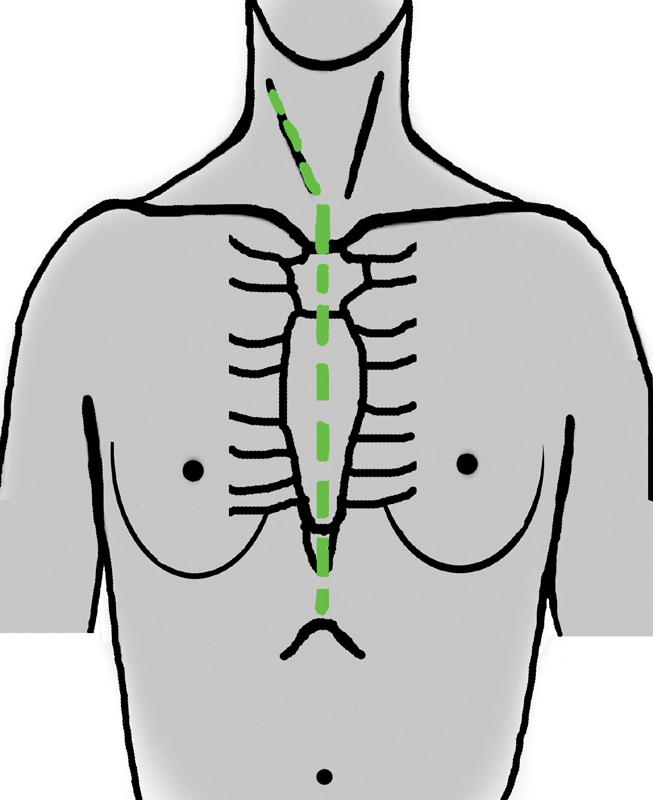
Sternocervicotomy incision.

After exposure via a right cervicosternotomy ( Figs. 2 and 3 ), RSA and right femoral artery cannulations were performed. During preparation of the arch and the large supra-aortic vessels, the patient was cooled down to 25°C. The ascending aorta was clamped and cardiac arrest was obtained with cold cardioplegia. After circulatory arrest, with maintained selective antegrade cerebral perfusion through the RSA, supra-aortic branches were clamped and the aortic arch incised. Close inspection showed a dilated and thickened aorta with a thin, thrombus-filled pouch at the base of the arch. Diseased tissues were resected, starting at the ascending aorta, including IA, proximal LCA and LSA, and the proximal descending aorta.

The aortic arch was replaced by a 28-mm Intergard Woven 4-branch Dacron graft. Anastomoses were performed in a retrograde fashion, starting from the descending aorta. After the descending aorta was connected to the graft, retrograde perfusion was restarted, suturing successively the LSA, RCCA, RSA, and the ascending aorta in an end-to-end fashion, with staged repetitive prosthetic cross-clamping after each anastomosis. The chronically occluded LCCA was not revascularized. Total cross-clamping time was 110 minutes.

**Fig. 4:**
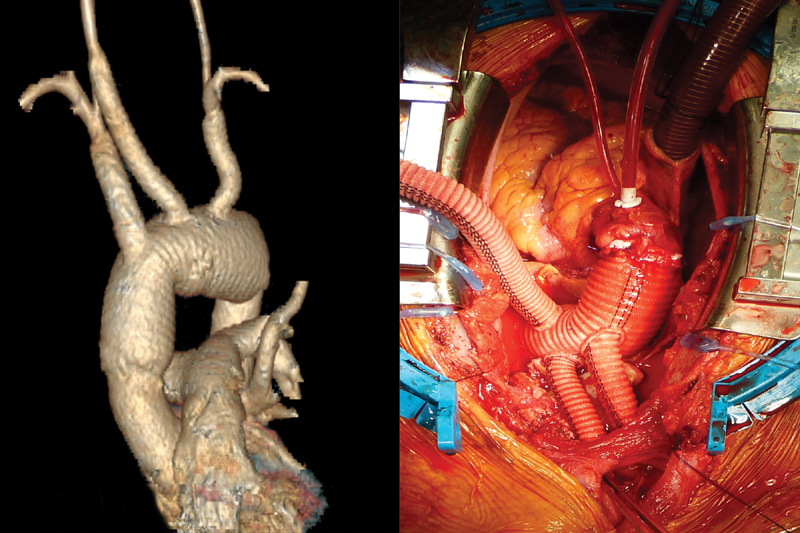
Postoperative computed tomography scan reconstruction (left). Operative view (right).

Postoperative CT excluded residual stenosis ( Fig. 4 ). Further postoperative evolution was uneventful. In particular, the neurological status was normal. Postoperative CT scan revealed no residual aneurysm or stenosis. The patient left hospital after 7 days. Histopathology revealed only unspecified inflammatory disease, as it is usual in this kind of pathology.

## Discussion

Cardiovascular involvement in RP is, with a reported incidence of 2 to 6%, uncommon, affecting mainly the aortic and mitral valves. 1 This complex systemic disease, involving multiple organs and tissues, may overlap frequently with other autoimmune diseases; therefore, diagnosis and correct classification of RP-mediated vasculitis remains difficult. 1 In the present case, despite the already known and documented RP, the appearance of the aortic inflammation, causing aneurysmal dilatation and stenosis, may be confounded with a Takayasu arteritis (TA). Histopathologically, both forms of aortitis present similar histologic patterns, that is, severe inflammation of the vasa vasorum. 2 3 Irrespective of the etiology, the indication for the surgical aortic arch replacement was given from the fast progression of the aneurysm (20 mm aneurysmal increase during the last 9 months), to a maximal dilatation of 65 mm. Performing preoperatively a fluorodeoxyglucose (FDG)-PET–CT allowed estimation of the localization of the quantitatively most important inflammatory activity of the vascular process. As already known for TA, restenosis rates are lower if anastomoses can be performed in “healthy” vessel sections. 3 4

Arterial involvement was mainly localized in two zones, first, within the very thin pouch in the inferior arch and, second, around the IA.

Perioperative observation of the former confirmed a very fragile zone of the arch, close to rupture. The association between FDG uptake in inflammatory vessel diseases and the risk of rupture is not well established and mainly based on animal models. 5 6

According to the present observation, the PET–CT is helpful in identifying vascular sections at risk.

An 8 to 31% restenosis rate at 3 to 6 years has been reported for TA, after an initial successful surgery; however, no results are available for RP. 3 For endovascular techniques, even higher restenoses rates (31% after 2 years, 57–78% after 10–12 years) have been reported. 3 4 7 One potential explanation may be due to the fact that an endoprosthesis will only cover (not extirpate) the diseased vessel, and therefore the inflammation can probably progress, contrary to the surgical approach with full resection and complete replacement of the most diseased segments of active inflammatory tissues. 3 7

RP is a rare etiology of aneurysm of the great vessels. In this context, a PET–CT is useful to evaluate the inflammatory activity (both intensity and location), indicating the risk of vessel rupture. The PET–CT data help to optimize the surgical approach. Surgical repair of an RP aortitis is safe, but close follow-up is mandatory to detect relapse.
